# COVID-19 and Dentistry: Enhancing Knowledge and Attitudes towards Infections, Immunity, and Vaccination

**DOI:** 10.3390/vaccines11071158

**Published:** 2023-06-26

**Authors:** Ajinkya M Pawar, Mohmed Isaqali Karobari

**Affiliations:** 1 Department of Conservative Dentistry and Endodontics, Nair Hospital Dental College, Mumbai 400008, Maharashtra, India; 2 Department of Restorative Dentistry & Endodontics, Faculty of Dentistry, University of Puthisastra, Phnom Penh 12211, Cambodia; 3 Department of Conservative Dentistry, Faculty of Dental Medicine, Universitas Airlangga, Surabaya 60131, East Java, Indonesia; 4 Center for Transdisciplinary Research, Saveetha Dental College, Saveetha Institute of Medical and Technical Science, Saveetha University, Chennai 600077, Tamil Naidu, India

**Keywords:** SARS-CoV-2, dental, dentistry, dental education, medical education, knowledge, vaccines, COVID-19, vaccine acceptance, vaccine hesitancy, COVID-19 vaccine, pandemic, prevention, transmission, immunity, dental clinics, dental anxiety, dental professionals, impact on practice, disease transmission

The COVID-19 pandemic has disrupted every part of human life, including healthcare systems, and presented societies with hitherto unheard-of concerns. Due to the nature of dental practice and its proximity to the respiratory tract, dentistry has been considerably influenced compared to other healthcare professions. The dental care community must be knowledgeable, proactive, and aware of new information regarding COVID-19, infections, immunity, and vaccines as we manage this problem [1,2]. This Special Issue seeks to illuminate the many facets of COVID-19 and dentistry, fostering a deeper comprehension of the difficulties encountered and offering perspectives on the best practices to reduce hazards.

Dental professionals now face new challenges due to COVID-19, forcing them to reaffirm their understanding of the virus and how it spreads. Implementing efficient infection control procedures in dental settings requires an understanding of the most recent scientific developments and recommendations [3]. By giving a thorough overview of COVID-19 and its implications for dentistry, this Special Issue strives to address the knowledge gap. We want to provide dental practitioners with the information they need to adjust their practices to the changing pandemic scenario through articles grounded in research and expert analysis.

When it comes to infection control, attitudes have a significant impact on behaviour, and dental workers are no exception. We hope to encourage a positive attitude change by emphasizing the need to follow safety guidelines, use personal protective equipment, and follow strict sterilization and disinfection procedures. In addition to protecting the dental team, stressing the value of maintaining a safe atmosphere gives confidence to people seeking dental care during these trying times [4].

The production and application of vaccines have become essential tools in our war against the virus as the world struggles to contain the COVID-19 epidemic. Dental professionals must be thoroughly aware of the immune response and the function of vaccinations in the fight against COVID-19 in order to practice dentistry [5]. In this special edition, we examine the facts underlying immunization and dispel common misconceptions.

The significance of a unified stance in the face of a global health catastrophe has been highlighted by COVID-19. Dental workers must keep abreast of the most recent advancements in COVID-19 research, infection control techniques, and vaccine tactics in order to practice dentistry effectively. This Special Issue is a tool to broaden understanding, mould attitudes, and inspire action. We can all help create a safer environment for patients and practitioners by arming dental professionals with evidence-based knowledge [6].

A fundamental change in knowledge, attitudes, and practices is now necessary as a result of the COVID-19 epidemic, which has completely altered the dental industry. The important elements of infections, immunity, and vaccination are highlighted in this Special Issue, which is focused on COVID-19 and dentistry. We can improve the safety and wellbeing of the dental team and their patients by educating dental workers on the relevant information and encouraging a positive attitude. Let us use this chance to fortify our community’s reaction to COVID-19 and pave the way to a healthier future.
